# Benefit-finding buffers the effects of quarantine on older adults’ mental health during the COVID-19 pandemic

**DOI:** 10.1093/geroni/igab046.2674

**Published:** 2021-12-17

**Authors:** Fan Zhang, Sheung-Tak Cheng

**Affiliations:** 1 Jinan University, Jinan University, Guangdong, China (People's Republic); 2 Education University of Hong Kong, The Education University of Hong Kong, Not Applicable, Hong Kong

## Abstract

**Objective:**

Older adults’ health and well-being may suffer due to prolonged social isolation leading to loneliness and increased stress during the COVID-19 pandemic. The current study aimed to address the role of benefit-finding, defined as the capacity to derive meaning and positive aspects from stressful situations, in older adults’ adaptation to the effects of quarantine.

**Methods:**

421 participants aged 50 or above in China participated in an online survey to study the effects of quarantine on loneliness, stress, anxiety, depression and life satisfaction, and the moderating role of benefit-finding.

**Results:**

The results showed that quarantine was basically unrelated to any outcome. Further analysis showed, however, that the effect of quarantine varied by levels of benefit-finding. Only people with lower benefit-finding reported a higher level of loneliness, perceived stress, anxiety and depression, but no relationships were found at higher benefit-finding.

**Conclusions:**

The findings extended our understanding of the role of benefit-finding in buffering the negative impact of adversity on older people. By mitigating the effects of prolonged social isolation, benefit-finding served as a protective factor in older people’s adaptation to the sequelae of this pandemic.

